# Nutrients and Bioactive Compounds in Peruvian Pacay (*Inga feuilleei* D.C.)

**DOI:** 10.3390/foods15020278

**Published:** 2026-01-12

**Authors:** Mario Cotacallapa-Sucapuca, Rosa M. Cámara, María Ciudad-Mulero, Genciana Serruto-Medina, Romualdo Vilca-Curo, Claudia Arribas, Mercedes M. Pedrosa, Patricia Morales, Montaña Cámara

**Affiliations:** 1 Departamento de Nutrición y Ciencia de los Alimentos, Facultad de Farmacia, Universidad Complutense de Madrid, Plaza Ramón y Cajal s/n, 28040 Madrid, Spain; mcotacallapas@unam.edu.pe (M.C.-S.); rosacama@ucm.es (R.M.C.); mariaciudad@ucm.es (M.C.-M.); patmoral@ucm.es (P.M.); 2 Escuela Profesional de Ingeniería Agroindustrial, Universidad Nacional de Moquegua, Prolongación Ancash s/n, Moquegua 18001, Peru; rvilcac@unam.edu.pe; 3 Escuela Profesional de Gestión Pública y Desarrollo Social, Universidad Nacional de Moquegua, Prolongación Ancash s/n, Moquegua 18001, Peru; gserrutom@unam.edu.pe; 4 Dpto. Tecnología de Alimentos, INIA-CSIC, Ctra. de La Coruña km 7.5, 28040 Madrid, Spain; arribas.claudia@inia.es (C.A.); mmartin@inia.csic.es (M.M.P.)

**Keywords:** natural resources, sustainability, pacay, Peruvian plant

## Abstract

Pacay (*Inga feuilleei* D.C.) is a species native to Peru. To the author’s knowledge, the only information found in the literature reference of the chemical composition of the pacay’s edible part (fruit) corresponds to the Peruvian table of food composition of the Ministry of Health. Considering the lack of information on this important plant, this study aims to (1) compare the nutritional profiles of the Limeña and Corriente varieties, (2) evaluate the distribution of bioactive compounds across the fruit’s principal fractions (pulp, seed, and peel/mesocarp), and (3) determine the antioxidant capacity and bioactives associated with each fraction. Results showed clear differences both between plant tissues and between genotypes. The edible part showed high amounts of carbohydrates (84–87%), seeds are rich in protein (18–21%), and peels are rich in fiber (around 34%). Amylopectin was the majority starch fraction (86%) found in pacay seeds. All pacay fractions stand out for their high content of total polyphenols, being higher in the case of the peel (1843 mg GAE/100 g). Hydroxycinnamic acids content (40–136 mg FAE/100 g) was higher than the flavonols (18–50 mg GAE/100 g), and both were present in higher amounts in the case of the seed fraction. These findings could be important to enhance the knowledge about this species and its revalorization as functional ingredients to be used in food formulation.

## 1. Introduction

Globally, there is an increasing interest in preserving seed diversity, which constitutes a staple source of food, as the consumption of vegetables is recognized worldwide as an important aspect to achieve a healthy diet [1,2]. The Food and Agriculture Organization of the United Nations has reported that numerous plant species and varieties that were used by ancestors for feeding are already extinct, pointing out that it is essential to ensure crop biodiversity to keep our food systems strong and resilient against these threats [3,4]. At this point, and in terms of biodiversity, Peru is recognized as a highly diverse nation, hosting an extensive array of plant species [5,6].

Pacay (*Inga feuilleei* D.C.) is a species native to Peru, which grows in the valleys of the highlands and coastal areas. Pacay was well used in Peru around 4500 B. C. [7]. It spread from its center of domestication during the Inca Empire, through Chile, Bolivia, and to Ecuador, expanding through South America and Central America. Consequently, pacay also refers to guaba species (Inga edulis) in Ecuador and Brazil, and Paqaya (Inga de macao) in Bolivia, and has been introduced to other areas of Central America and other places, including Australia and Malaysia [8]. It belongs to the Inga genus, from the Leguminoseae family (Fabaceae Lindl), one of the most important species ecologically and for humans due to its multiple uses [9]. The genus Inga Mill. comprises approximately 300 species with a Neotropical distribution [10,11,12] and it is represented by approximately 180 species, of which only a few have appreciable quality as edible, fresh fruit. Among these appreciated species, Inga edulis is one of the most interesting, as it has been significantly improved through cultivation [13,14].

The pacay tree is distinguished by its arboreal size. It has paripinnate leaves, glands, and nectar located between each pair of leaflets. Its fruits are fleshy and indehiscent, and they are located in pods. The pacay contains an edible white fruit that covers the seed [8] or is yellowish brown in color. The seeds are embryos with flat convex, thick, hard, invaginated, split, and cryptoradicular cotyledons of generally dark colors; the surface is smooth and shiny [15]. The seeds, three to four, are embedded in the edible part, which corresponds to a white and sweet cottony pulp [8]. The shell is discarded during consumption, without further use [12,16,17,18]. Thus, the edible part of the pacay fruit is the sarcotesta that surrounds the seed through flat, whitish funicles that can be easily detached and have a moist, whitish, hairy, fleshy, tender, and dense consistency [15].

Peru has around 87 species of the Inga genus [16,18], highlighting the species Inga edulis Mart. and *Inga feuilleei* D.C., characterized by cylindrical and quadrangular pods, respectively [19]. It is known that the cultivation of traditional Andean crops was essential for the feeding of ancient populations, as this has been revealed by the study of the presence of starch grains on human teeth, indicating an early broad crop diet in northern Peru. In particular, starch from the seeds of *Inga feuilleei* was routinely present on numerous teeth that dated between 8210 and 6970 14C yr B.P. [7]. Nowadays, the production of *Inga feuilleei* in the Moquegua region, with an average of 132.75 tons [20], is one of the activities that generates economic income for families dedicated to its cultivation, being important to enhancing the knowledge about this species and revalorizing it. The shrub that contains the pacay fruit is widely cultivated by the population to produce edible fruits, firewood, and as a shade tree. As an agroforestry component, it requires little phytosanitary control.

The pacay tree is useful to combat forest fragmentation or serve the ecological restoration of riparian forests due to its high resistance to adverse conditions, such as acidic soils and excess exposure to light [21,22]. Its high biomass production helps control weeds and erosion in alley crops and other agroforestry systems [23]. They are considered shade trees, used for coffee and cocoa crops in Mesoamerica [24]. Moreover, they are nitrogen fixers, capable of mitigating climate change with a potential positive effect on carbon sequestration in the soil [25] and contributing to the growth of other plant species [26].

The pacay fruit is sold in markets at low prices; consumers acquire the product for the organoleptic characteristics (sweetness, flavor, and color) of the fruit (edible part), and its beneficial health properties are unknown. The method of consumption is opening the pod where the seeds are covered in cottony pulp, and then discarding the seed and pod or shell, which constitute the largest proportion of the fruit.

In addition to its interesting properties at the environmental level, the pacay leaves are particularly rich in polyphenols, mainly flavonoids, and they have been used in popular medicine as an anti-inflammatory, nasal decongestant, antidiarrheal, and against arthritis, rheumatism, and digestive problems [26,27,28]. Regarding seeds, it has been reported that they have trypsin inhibitors, which offer protection to the fruit against insect damage, contributing to the development of biotechnological tools with greater resistance to insect pests [29,30,31].

To date, there are no substantial studies related to the bioactives or nutritional composition of pacay fruits, except for the study developed for a specific species (*Inga laurina* (Sw.) Willd.), where the proximal analysis, total phenolics, antioxidant activity, and mineral composition were determined [32]. The Inga edulis species contains functional properties, such as phenolics, flavonoids, antioxidant capacity [33], and fiber [34], whose components contribute to health benefits such as control of type 2 diabetes and hypertension [14]. Considering that the edible part is limited to the consumption of the cotton-like pulp (sarcotesta), the pod and seeds could be revalorized if they are used as by-products, taking advantage of their potential bioactive properties [35].

To the author’s knowledge, the only information found in the literature reference on the chemical composition of the edible part (fruit) of pacay (*Inga feuilleei*) corresponds to the Peruvian table of food composition of the Ministry of Health [36] with the terminology pacay or guaba.

Considering the lack of information on this important native Peruvian plant, this study is aimed at characterizing three different fractions (fruit, seed, and peel) from two pacay (*Inga feuilleei* D.C.) varieties, “Limeña” and “Corriente”, by determining the nutritional composition, carbohydrate and mineral fraction, some bioactive compounds, and antioxidant capacity.

## 2. Materials and Methods

The fruits of pacay (*Inga feuilleei* D.C.) varieties “Limeña” and “Corriente” were acquired in the central market of the city of Moquegua in Peru. Fruits were weighed and unsheathed to separate the sarcotesta (fruit edible fraction, sweet aril), seeds, and peel (mesocarp). To obtain a more stable sample, the three fractions were dried in an oven (Memmer UN55) at 40 °C (until constant weight) and pulverized (Grondoy GR-PV50B Mills, Lima, Perú). The dried samples were packaged in low-density polyethylene bags with a zip-lock closure, intended for proximate analysis. All analyses were conducted in triplicate.

The codes used for sample identification as well as their characteristics are shown in Table 1.

### 2.1. Proximate Analysis

Proximates were determined according to the standard methods of the Association of Official Analytical Chemists [37]. The fat content was determined by gravimetry using the Soxhlet method employing petroleum ether as extraction solvent. The total fiber content was determined by acid (H_2_SO_4_ 1.25% *w*/*v*) and alkaline (NaOH 1.25% *w*/*v*) digestion. The ash was determined by incineration at 600 °C until white ashes were obtained, indicating the organic material destruction. Humidity was determined in an oven at 105 °C until constant weight. The protein was analyzed by the Kjeldahl method through digestion with concentrated H_2_SO_4_, distillation with NaOH of appropriate concentration, and the distillate was received in 4% boric acid plus. Distilled water was titrated with 0.3 N HCl until a change in color, using methyl red as the indicator. The total carbohydrate content was determined by subtracting from 100 the values of protein, lipid, ash, and moisture [38]. The results are expressed in g/100 g dry weight (dw).

### 2.2. Soluble Sugars and α-Galactosides

**Figure 1:**
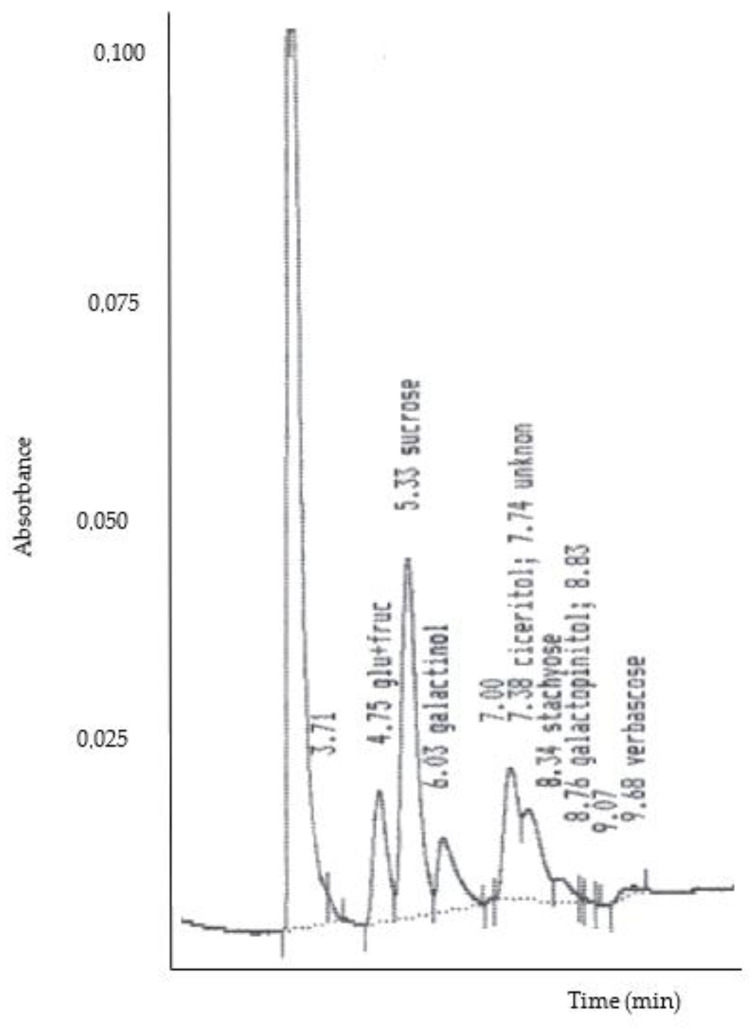
Representative HPLC-RI chromatogram of soluble sugars and α-galactosides, including sucrose, raffinose, ciceritol, stachyose, galactopinitol, and verbascose.

For sugar extraction, 100 mg of each sample was homogenized in 5 mL of aqueous ethanol (50% *v*/*v*) for 1 min using an Ultra-Turrax homogenizer (IKA^®^ Works, Inc., Wilmington, NC, USA); then, the mixture was centrifuged for 10 min × 10,000 rpm and the supernatant was decanted (this procedure was repeated twice). Supernatants were combined, filtered through Sep-Pak C18 cartridges (Waters, Milford, MA, USA), and evaporated to dryness. The residue was dissolved in 1 mL of double-deionized water and centrifuged for 10 min × 10,000 rpm. Prior to injection into the HPLC, samples were filtered through a 0.45 µm Millipore membrane. Then, soluble sugars and α-Galactosides were determined by liquid chromatography-RI using a Beckman HPLC System Gold (Los Angeles, CA, USA) equipped with a refractive index detector. A Spherisorb-5-NH2 column (250 × 4.6 mm i.d., Waters, Milford, MA, USA) was used with acetonitrile/water 60:40 (*v*/*v*) as a mobile phase at a flow rate of 1 mL/min, according to the methodology reported by Pedrosa et al. (2012) [39]. Sugars were quantified by comparing them to a calibration curve for sucrose, raffinose, ciceritol, stachyose, galactopinitol, and verbascose (Sigma, St. Louis, MO, USA). A linear response was observed in the range of 0–4 mg/ ml, with an R^2^ of 0.99. The curves obtained were as follows: Sucrose (y = 0.193x − 0.001), galactinol (y = 0.227x − 0.014), raffinose (y = 0.193x + 0.003), ciceritol (y = 0.295x + 0.030), and stachyose (y = 0.217x − 0.008). The tests were performed in triplicate, and results were expressed in g/100 g dw sample. A representative HPLC-RI chromatogram of soluble sugars and α-galactosides is shown in Figure 1.

### 2.3. Starch, Amylose, and Amylopectin

Total and resistant starch content in pacay fractions was analyzed using a Megazyme kit (Wicklow, Ireland). This kit contains an improved α-amylase that allows starch hydrolysis with amylase incubations at pH 3.8. Resulting glucose was determined by spectrophotometry at 510 nm according to Arribas et al. (2019) [40] based on the AOAC methods 996.11 and 76.13.01.

Amylose and amylopectin were determined according to Guzman et al. (2011) [41]. The percentage of amylose was calculated based on the amylose calibration curve reading at 620 nm and 510 nm in a spectrophotometer (Thomas Sicentific, Eppendorf BioSpectrometer basic, Swedesboro, NJ, USA), (y = 75.24x − 0.1163, R^2^ = 0.9942), and the percentage of amylopectin was the difference (100 − amylose).

### 2.4. Micronutrients: Vitamin C and Minerals

**Figure 2:**
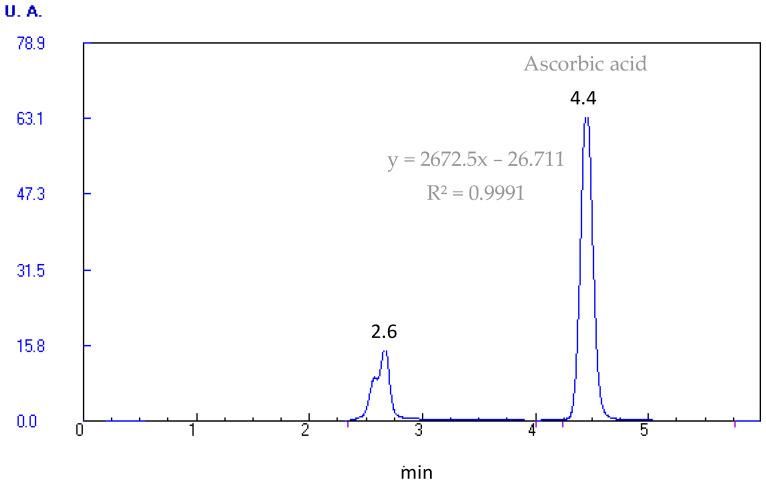
Representative HPLC-UV chromatogram of ascorbic acid, and equation of standard curve.

The extraction of vitamin C (L-ascorbic acid) was performed in acid medium [42]. Subsequent identification and quantification were completed by reverse-phase high-performance liquid chromatography (HPLC) with ultraviolet (UV) detection. Samples (0.5 g) were extracted with metaphosphoric acid 4.5% (*w*/*v*). The mixture was shaken in a magnetic stirrer (P-Selecta, Barcelona, Spain) for 15 min and kept from light. The extract was filtered with an Albet paper filter No. 1242. After that, a small aliquot of the extract was filtered through a 0.45 μm polyvinylidene fluoride membrane filter (Millex) and a specific volume was injected in HPLC for the quantification of vitamin C. The chromatographic equipment (Micron Analytical, Wilmington, DE, USA) incorporates an isocratic pump model PU-II, automatic injector AS-1555 (model Jasco), UV–visible detector (Thermo Separation Spectra Series UV100), and column ODS Sphereclone (2) (250 × 4, 60 mm; 5 μm). The mobile phase was H_2_SO_4_ (1.8 mM, pH 2.5–2.6). A flow rate of 0.9 mL/min and UV detection at 245 nm was used. The data obtained were analyzed with the software Biocrom 2000, 3.0, by comparison of the retention times of each chromatographic peak with those of standard products (Figure 2). The results were expressed in mg vitamin C/100 g sample.

Analyzed minerals (Na, K, Ca, Mg, Cu, Fe, Mn, Zn, and Se) were determined by Atomic Absorption Spectroscopy using ICP-MS as the detector. Results were expressed in mg each mineral/100 g dw sample.

### 2.5. Total Phenolic Compounds and Phenolic Families

Total phenolic compounds and phenolic families (hydroxycinnamic acids and flavonols) were analyzed by QUENCHER (QUick, Easy, New, CHEap, and Reproducible) methodology [43,44]. This procedure consists of submitting a small amount of the sample, previously homogenized until a particle size of 0.037 mm, to direct contact with the reagents of each assay. It allows the quantification of both soluble and insoluble compounds; thus, it is possible to obtain a more accurate and reliable result.

#### 2.5.1. Analysis of Total Phenolic Compounds by QUENCHER Methodology

The determination of total phenolic compounds was carried out by Folin–Ciocalteu assay, according to del Pino-García et al. (2015) [43]. This method is based on the capacity of the samples to reduce the Folin–Ciocalteu reagent. Briefly, 10 mg of each sample was weighted in triplicate, and 0.2 mL of distilled water, and 0.2 mL of Folin–Ciocalteu reagent were added, and mixtures were homogenized by vortex and put in an orbital shaker for 5 min. After that, 4 mL of Na_2_CO_3_ and 5.6 mL of distilled water were added. This was homogenized by vortex, and after 50 min of orbital shaking, samples were centrifuged for 5 min at 6500 rpm and filtered. The absorbance was measured at 750 nm in a UV–vis spectrometer, synergy HTX, Biotek. The results obtained were expressed in milligrams of gallic acid equivalent per 100 g of sample (mg GAE/100 g), as a calibration curve was obtained by reaction of different concentrations (50–400 μg/mL) of the standard.

#### 2.5.2. Analysis of Total Hydroxycinnamic Acids by QUENCHER Methodology

The determination of hydroxycinnamic acids (HCCs) was carried out by following the procedure described by Bonoli et al. (2004) [45]. In brief, 20 mg of each sample was weighted and mixed with 0.5 mL of distilled water and 4 mL of methanol. They were homogenized with a vortex and incubated in an orbital shaker for 15 min. After that, samples were centrifuged at 6500 rpm for 5 min and filtered. The absorbance was measured at 320 nm in a UV–Vis spectrometer, synergy HTX, Biotek. All the determinations were performed in triplicate. The results were calculated as milligrams of ferulic acid equivalent per 100 g (mg FAE/100 g, fw), as a calibration curve was prepared by reaction of different concentrations (3.125–200 μg/mL) of the standard.

#### 2.5.3. Analysis of Total Flavonols by QUENCHER Methodology

The analysis of flavonols (FC) was adapted from the methodology proposed by Bonoli et al. (2004) [45] and modified by Vega et al. (2023) [44]. In short, 0.5 mL of distilled water and 4 mL of methanol were added to 20 mg of each sample, weighted in triplicate. These mixtures were homogenized by vortex and incubated at room temperature for 15 min using an orbital shaker. The samples were centrifuged for 15 min at 6500 rpm and filtered. Finally, the absorbance was measured at 370 nm with a UV–Vis spectrometer, synergy HTX, Biotek. A calibration curve of quercetin was obtained with different concentrations of the standard (3.125–250 μg/mL). The results were expressed in milligrams of quercetin equivalent per 100 g (mg QE/100 g dw).

### 2.6. Antioxidant Capacity

The antioxidant capacity was evaluated by using an in vitro direct method (QUENCHER procedure) applied to DPPH methodology. The scavenging capacity of the 2,2-diphenyl-1-picriylhydrazyl (DPPH) radical by the samples was performed according to del Pino-García et al. (2015) [43]. Concisely, 15 mg was weighted by triplicate and homogenized with 10 mL of DPPH reagent by vortex. It was incubated for 1 h using an orbital shaker. Then, samples were centrifuged for 5 min at 7000 rpm and filtered. The absorbance was measured at 517 nm in a UV–vis spectrometer, synergy HTX, Biotek. The results were expressed as milligrams of Trolox equivalent per 100 g of sample (mg TE/100 g), and a calibration curve was prepared by reactions of different concentrations (6.25–400 μg/mL) of the standard.

### 2.7. Statistical Analysis

Mean ± standard deviations (SDs) were determined, and the data was statistically analyzed by analysis of variance (ANOVA). Duncan’s test was applied to identify the differences between varieties or fruit fractions. All statistical analyses were performed using Statgraphics Plus 5.1 software. The statistical significance level was set at *p* < 0.05.

## 3. Results and Discussion

### 3.1. Proximate Analysis Results

The results obtained from the proximal composition analysis are shown in Table 2.

From a nutritional point of view, legumes are very interesting foods as they are rich in proteins, carbohydrates, and dietary fiber [46]. As expected, the proximate composition between the different pacay fractions is different. Furthermore, sarcotesta (sweet aril, edible) of the Corriente and Limeña varieties were significantly different (*p* < 0.05), with the Corriente variety having higher moisture, ash, protein, and crude fiber compared to the Limeña variety (Table 2). The moisture (9.61 and 7.66 g/100 g, Corriente and Limeña, respectively) and carbohydrates (83.98 and 86.45 g/100 g, Corriente and Limeña, respectively) of the fruit were significantly higher than the ones found in the seed and mesocarp in both varieties, while ash, fat, and protein were significantly lower in both varieties. The carbohydrate content is the most notable fraction in the fruit of both varieties. It is known that insufficient consumption of fiber in Western societies is directly linked to certain diseases, and legumes are good sources of fiber [47,48]. In this study, peel pacay fraction stands out for its high fiber content (more than 30 g/100 g dw), while fiber values of the edible fruit were relatively close to those reported in the *Inga lauriana* variety of 4.5 g/100 g [32].

The proximate composition of the seed was significantly different between the Corriente and Limeña varieties, except for fiber, which did not show significant differences, with the Corriente variety having higher humidity, ash, and carbohydrates and lower fat and protein than the Limeña variety (Table 2). Fat, protein, and even caloric energy are significantly higher with respect to the fruit and mesocarp in both varieties, as occurs in other varieties such as Inga lauriana [32] and Inga-açu [49]. The amount of protein (18.15 and 20.52 g/100 g, Corriente and Limeña, respectively), is also highlighted in another study with a relatively similar value such as Inga lauriana (19.52 g/100 g) [32]. Water content values (humidity) were above 5% on a dry basis. Seeds of these species are recalcitrant in nature; that is, they do not tolerate desiccation to ≤5% moisture content, which can make their storage difficult for long periods of time or at low temperatures [15,50].

The peel (mesocarp) used in the present study corresponds to the internal layers of the pacay pod. The proximal composition of the mesocarp was significantly different between the Corriente and Limeña varieties, except for fat and fiber content, which did not show a significant difference between both varieties (Table 2). Ash and fiber were significantly higher compared to fruit and seed in both varieties. No studies were found on the specific proximal composition of the mesocarp, although they did mention the proximal composition of the shell [49], so it is not known whether they refer to the mesocarp or the pod as a whole. In another study, they verified that the morphological length of cultivated pods was greater than wild ones in the Peruvian Amazon [51], which could imply differences in the species cultivated in coastal areas, hence their proximal composition.

All these results could suggest that the seeds and even the pods have great potential as food for human use, either directly or as a food ingredient in the future.

### 3.2. Carbohydrate Fraction: Sugars and Oligosaccharides, Starch, Amylose and Amylopectin

Regarding carbohydrate fraction, sugar and alpha-galactoside content were determined in both varieties (Limeña and Corriente) in the three fractions studied: peel, seed, and fruit (aril). Sucrose was the main sugar present in the three fractions, being predominant in the fruit, which corresponds to the white sweet pulp surrounding the seed, and has given the pacay its name as the Peruvian ice cream bean.

Oligosaccharides and non-starch polysaccharides are quite interesting compounds in preventing and treating functional gastrointestinal disorders [52]. The main oligosaccharides, alpha-galactosides, present in legumes are stachyose, raffinose, and verbascose; in addition, ciceritol could also be found [53]. FODMAPs (Fermentable Oligosaccharides, Disaccharides, Monosaccharides, and Polyols) are poorly digested short-chain carbs that draw water and ferment in the large intestine, causing gas, bloating, pain, and altered bowel habits. Their digestibility is low because the body lacks enzymes or transporters to absorb them fully in the small bowel, so they reach the colon where gut bacteria rapidly ferment them, producing gas and osmotic effects. At the same time, these compounds have a beneficial prebiotic effect [54].

In this study (Table 3), the pacay fruit edible fraction from the Limeña variety did not contain detectable α-galactosides, whereas the Corriente variety presented measurable levels of raffinose. In the seeds, both varieties exhibited similar concentrations of raffinose; however, the Limeña variety additionally contained stachyose—absent in the Corriente variety—and showed a higher abundance of verbascose. Marked differences were observed in the peel, likely attributable to compositional and morphological differences between the varieties. Only the Limeña peel contained raffinose and stachyose, and although verbascose was present in both varieties, its concentration in the Limeña peel was nearly twice that of the Corriente variety.

Both in the seed and peel fraction, an unknown pick (quantified using the raffinose calibration curve) was found, which does not correlate with any of the available standards. To our knowledge, no prior literature describes the α-galactoside composition of *Inga feuilleei* (pacay) fruit or its Peruvian varieties, preventing direct comparison with other studies.

As shown in Table 4, in terms of total starch content, as expected, it was mainly found in the seeds of both varieties, being slightly lower in the Limeña variety. However, the Limeña variety has a higher starch content in the fruit and contains starch in the peel, unlike the Corriente variety. Resistant starch has only been detected in the seeds of both varieties and in very similar quantities. Analysis of the amylose and amylopectin fractions in the seed, which had the highest starch content, revealed a similar proportion in both varieties.

### 3.3. Micronutrients: Vitamin C and Minerals

Vitamin C is essential for humans, and it has important physiological functions, such as antioxidant compounds and enzyme cofactors, among others [55]. Even though it has interesting properties as a bioactive compound, vitamin C content does not stand out in Andean legumes, including pacay. As shown in Table 5, vitamin C was present in modest amounts in seed of both studied varieties, and it was also found in the fruit of the Limeña variety. The content of this micronutrient was significantly (*p* < 0.05) higher in sample PL#S.

Studies about the content of vitamin C in eight different legumes traditionally consumed in the Andean regions of Ecuador, namely Chocho, Chocho perla, Frejol blanco, Frejol Canario, Frejol de castilla, Frejol negro, Frejol panamito, and Haba Pallar, showed that vitamin C was only found in Haba Pallar (15.53 mg of vitamin C per 100 g) [56].

**Figure 3:**
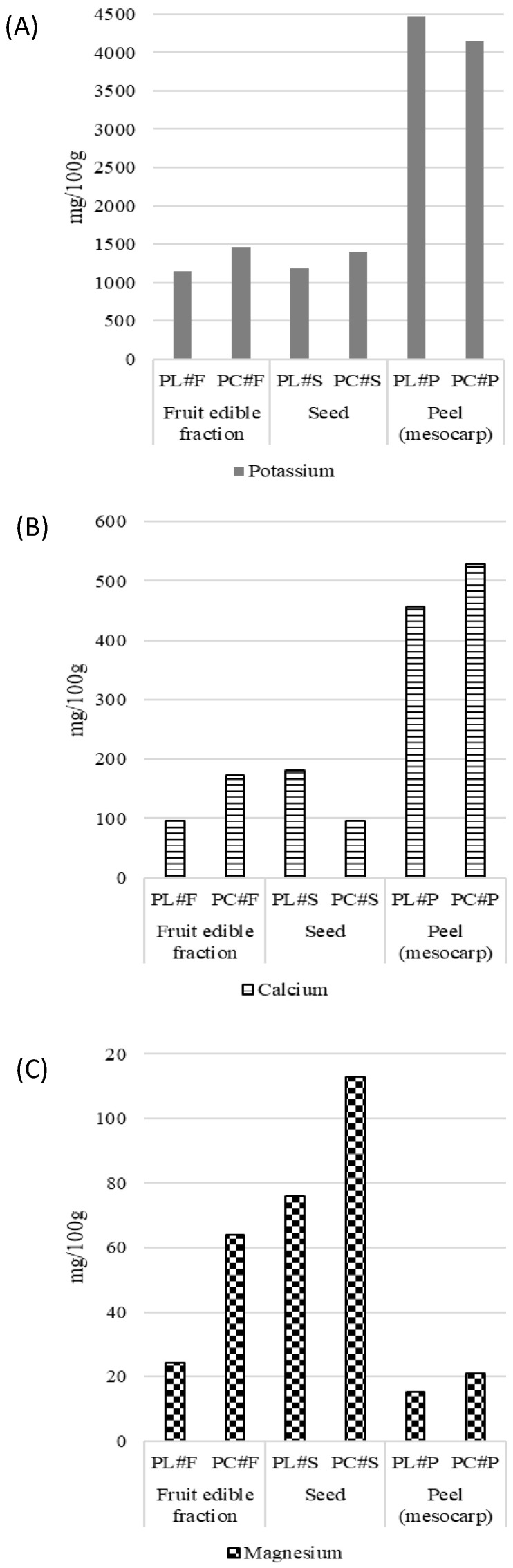
Macroelement content in pacay samples (mg/100 g dw, mean ± SD). (**A**): Potassium, (**B**): Calcium and (**C**): Magnesium.

**Figure 4:**
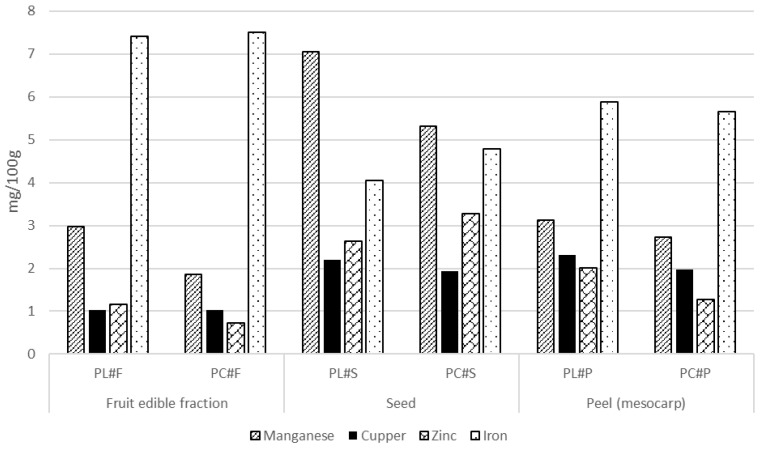
Microelement content in pacay samples (mg/100 g dw, mean ± SD).

**Figure 5:**
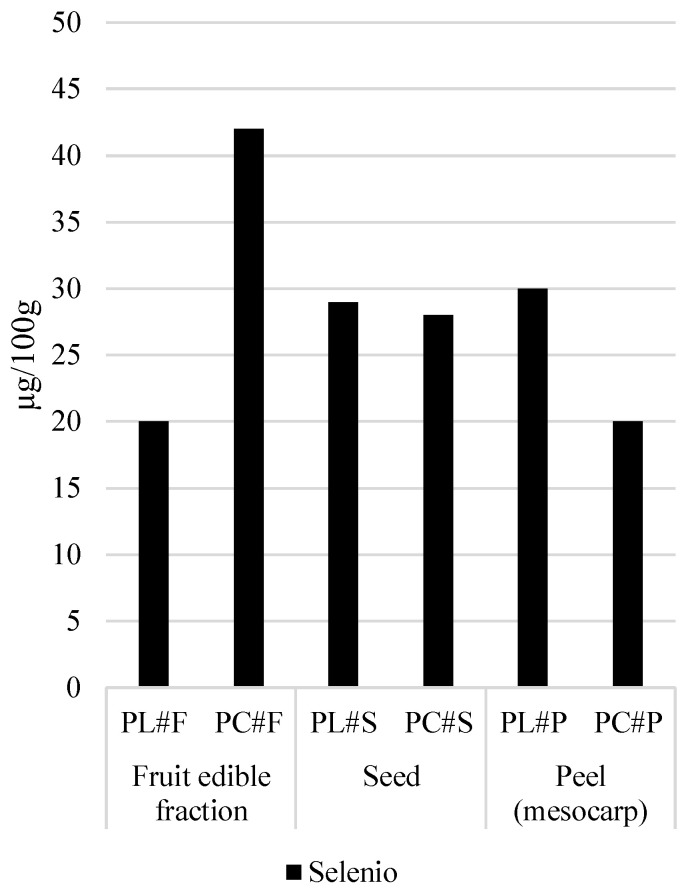
Selenium content in pacay samples (µg/100 g dw, mean ± SD).

Essential elements are indispensable for the proper functioning of the body, as they carry out vital functions. These minerals include sodium, potassium, calcium, magnesium, molybdenum, manganese, copper, iron, zinc, chromium, and cobalt, and they can be found naturally in food. Depending on the required amounts, they are subdivided into two groups: macroelements, which are needed in large quantities, and trace elements (or microelements), which, although required in small amounts, are just as important as the former. The results obtained from the mineral composition analysis are shown in Figure 3, Figure 4 and Figure 5.

Macroelements (potassium, calcium, and magnesium) are shown in Figure 3. Potassium, the most abundant mineral detected, showed a similar trend in the edible fruit fraction and seed, but Limeña surpassed Corriente in peel potassium, suggesting that K partitioning in Limeña may favor peripheral tissues. The Corriente variety showed greater Ca and Mg accumulation in the fruit edible fraction and peel, while Limeña only exceeded Corriente in seed Ca content. Sodium was reported below quantification limits (<15 mg/mL) in all samples, indicating minimal Na accumulation in both varieties, which is a nutritionally favorable characteristic of these pacay fruit fractions.

The Limeña variety was richer in several trace minerals, including iron, manganese, and zinc, particularly in the fruit edible fraction, which is of dietary relevance. As seen in Figure 4, Manganese and zinc followed parallel patterns, with Limeña presenting consistently higher levels in the fruit edible fraction and peel, although Corriente seeds showed higher Zn concentrations. Selenium concentrations were low across pacay fruit fractions, with only modest varietal differences. While Selenium concentrations were generally higher in the Corriente edible fruit fraction, the levels remained nutritionally negligible (values ranged from 20 to 30 μ/100 g), as seen in Figure 5.

The pacay peel fraction stands out as a mineral-rich tissue for both varieties, especially for potassium and calcium, reinforcing its potential value in food fortification or functional ingredient development.

### 3.4. Bioactives: Total Phenolic Compounds, Phenolic Families, and Antioxidant Activity

Hydrosoluble antioxidants and other compounds, which exert antioxidant properties, previously reported in legumes could lead to important health benefits [46,57]. The results obtained from the analysis of phenolic composition (total phenolic compounds and phenolic families) are shown in Table 6.

Total phenolic compounds in analyzed fractions from pacay (Limeña and Corriente varieties) ranged between 204.31 and 1843.39 mg GAE/100 g in samples PL#S and PL#P, respectively. The obtained values were higher than those previously published by Cálamo Benza (2014) [58] but are in the range previously reported for different legumes consumed in the Andean regions of Ecuador, in which total phenolic content varies between 80.19 and 1270.75 mg Equivalents of gallic acid/100 g [58]. Statistically significant differences (*p* < 0.05) were observed, with total phenolic content being higher in the peel fraction, while the lower concentration was found in the case of seeds.

Regarding phenolic families, the content of hydroxycinnamic acids was higher than the flavonol concentration, and both were present in higher amounts in the case of the seed fraction (Table 6). Peel mesocarp stands out with the high total phenolic content in comparison with other fruit fractions. In the edible fruit fraction, differences between varieties were only found with total phenolic content being higher in the Corriente variety.

All pacay fractions stand out for their high content of total polyphenols, the content of these being clearly higher in the case of the peel (1843.3 ± 0.83 mg Eq. of gallic acid/100 g in the Lima variety and 1394.82 ± 1.16 mg Eq. of gallic acid/100 g in the Corriente variety). Next is the fruit (323.55 mg Eq. of gallic acid/100 g in the Limeña variety and 558.81 mg Eq. of gallic acid/100 g in the regular variety) and finally the seed. These values are higher than those previously published by Cálamo (2014) [58]. Furthermore, it stood out for its high content of hydroxycinnamic acids compared to flavonols, with the content in the seeds of both varieties predominating. Pacae peel also has a higher antioxidant capacity than the seed and pulp, as evaluated using the DPPH technique.

These results are consistent with those reported by Calamo (2014) [58] using FRAP and ABTS+ techniques and results from other Inga species, as reported by Silva et al. (2007) [34], which measure the antioxidant capacity (ABTS assay) of Inga edulis in fresh weight: leaves: 58.1 ± 44.9 μg TE/mg, peel: 31.4 ± 1.8 μg TE/mg, fruit (pulp): 2.2 ± 0.4 μg TE/mg, and seed: 1.0 ± 0.2 μg TE/mg. Also in studies on the properties of Inga edulis conducted by Tauchen et al. (2016) [33], the antioxidant capacity (ABTS assay) was reported as follows: epicarp: 20.8 ± 1.8 μg Trolox equivalent (TE)/mg extract; leaves: 262.3 ± 11.8 μg TE/mg extract, pericarp: 207.2 ± 13.8 μg TE/mg extract, and seed: 17.2 ± 3.3 μg TE/mg extract.

Martins et al. (2014) [32], in a different species of Inga genus, namely Inga laurina, studied the total phenolic content. These authors also reported that the main concentration of polyphenols was found in the peel fraction.

The present study has two limitations, one related to the pacay samples considered in this study that were acquired in the central market of the city of Moquegua in Peru, which may add variability, and second, the scarce scientific data available to compare our results properly.

## 4. Conclusions

This study demonstrates that pacay, *Inga feuilleei,* is a rich source of nutrients and bioactive compounds, with differences between varieties and among fruit fractions. The peel, in particular, shows exceptional levels of polyphenols and antioxidant capacity, suggesting its value as a functional ingredient.

Pacay peel—currently treated as waste—represents a promising functional ingredient due to its high antioxidant capacity. Its incorporation into food matrices (e.g., bakery products, beverages, dietary supplements) could support the development of novel functional foods, such as everyday foods like snacks, bakery, dairy, and beverages, to improve texture and functionality, contributing to waste reduction and circular economy strategies. Thus, the valorization of pacay by-products could promote sustainable food innovation and increase the economic relevance of this underexplored Andean fruit.

## Figures and Tables

**Table 1:** Code and description of pacay *Inga feuilleei* D.C. fruit fractions analyzed.

Sample *Inga feuilleei* D.C.	Sample Fraction	Sample Code
Limeña variety	Fruit edible fraction	PL#F
	Seed fraction	PL#S
	Peel fraction (mesocarp)	PL#P
Corriente variety	Fruit edible fraction	PC#F
	Seed fraction	PC#S
	Peel fraction (mesocarp)	PC#P

**Table 2:** Proximate analysis results from dried pacay fractions: fruit edible fraction, seeds, and peel (g/100 g dw, mean ± SD).

	Sample					
	Fruit Edible Fraction		Seed		Peel (Mesocarp)	
Variety:	Limeña	Corriente	Limeña	Corriente	Limeña	Corriente
Code:	PL#F	PC#F	PL#S	PC#S	PL#P	PC#P
Moisture	7.66 ± 0.41 a	9.61 ± 0.11 b	5.24 ± 0.11 a	7.29 ± 0.13 b	6.37 ± 0.05 b	5.90 ± 0.04 a
Carbohydrates	86.45 ± 0.51 b	83.97 ± 0.08 a	71.21 ± 0.05 a	71.54 ± 0.22 b	74.55 ± 0.18 b	72.37 ± 0.11 a
Fiber	3.30 ± 0.08 a	3.99 ± 0.11 b	2.18 ± 0.19 a	2.30 ± 0.09 a	34.85 ± 0.02 a	34.23 ± 0.02 a
Fat	0.18 ± 0.01 b	0.14 ± 0.01 a	0.55 ± 0.03 b	0.46 ± 0.04 a	0.34 ± 0.01 a	0.33 ± 0.02 a
Protein	3.97 ± 0.07 a	4.29 ± 0.15 b	20.52 ± 0.11 b	18.15 ± 0.25 a	12.91 ± 0.13 a	11.73 ± 0.09 a
Ash	1.74 ± 0.03 a	1.98 ± 0.00 b	2.48 ± 0.01 a	2.56 ± 0.04 b	5.83 ± 0.01 a	6.60 ± 0.03 b

Dw: dry weight. Different letters mean statistically significant differences (*p* < 0.05) compared by the Tukey HSD test, between varieties for each parameter and fruit fraction analyzed.

**Table 3:** Sugar and oligosaccharide (g/100 g dw) content in pacay samples (mean ± SD).

Sample	Sucrose	Galactinol	Raffinose	Ciceritol	Unknown *	Estaquiose	Galactopinitol	Verbascose
	Fruit edible fraction							
PL#F	41.63 ± 1.31	0.96 ± 0.05	0.00 ± 0.00	0.00 ± 0.00	0.00 ± 0.00	0.00 ± 0.00	0.00 ± 0.00	0.00 ± 0.00
PC#F	36.92 ± 2.47	0.97 ± 0.17	0.42 ± 0.00	0.50 ± 0.00	0.00 ± 0.00	0.00 ± 0.00	0.00 ± 0.00	0.00 ± 0.00
	Seed							
PL#S	2.54 ± 0.01	0.00 ± 0.00	0.11 ± 0.02	1.48 ± 0.01	2.65 ± 0.07	0.34 ± 0.02	0.49 ± 0.00	0.92 ± 0.06
PC#S	2.72 ± 0.01	0.00 ± 0.00	0.11 ± 0.01	0.91 ± 0.04	2.96 ± 0.25	0.00 ± 0.00	0.04 ± 0.00	0.19 ± 0.01
	Peel, mesocarp							
PL#P	0.58 ± 0.12	0.96 ±0.09	0.22 ± 0.01	4.60 ± 0.11	4.64 ± 0.24	0.95 ± 0.07	0.13 ± 0.01	0.64 ± 0.04
PC#P	4.11 ± 0.15	0.88 ± 0.02	0.00 ± 0.00	2.00 ± 0.05	1.26 ± 0.03	0.00 ± 0.00	0.00 ± 0.00	0.39 ± 0.02

* Unknown pick was quantified using the raffinose standard. Dw: dry weight.

**Table 4:** Starch (g/100 g dw) content in pacay samples and proportion of amylose and amylopectin found in seeds (mean ± SD).

	Sample	Starch (g/100 g)	Resistant Starch (g/100 g)	Amylose (%)	Amylopectin (%)
Fruit edible fraction	PL#F	1.86 ± 1.03 b	NQ	ND	ND
	PC#F	0.57 ± 0.60 a	NQ	ND	ND
Seed	PL#S	40.29 ± 1.59 a	2.33 ± 0.24 a	13.48 ± 0.17 a	86.52 ± 0.17 a
	PC#S	47.20 ± 0.81 b	2.31 ± 0.24 a	13.93 ± 0.31 a	86.07 ± 0.31 a
Peel, mesocarp	PL#P	0.34 ± 0.12 b	NQ	ND	ND
	PC#P	0.00 ± 0.00 a	NQ	ND	ND

NQ = non-quantified, ND = non-determined. In each column, different letters mean statistically significant differences (*p* < 0.05) compared by the Tukey HSD test.

**Table 5:** Vitamin C content in the analyzed pacay samples (mg/100 g dw, mean ± SD).

	Sample	Vitamin C (mg/100 g)
Fruit edible fraction	PL#F	3.59 ± 0.04
	PC#F	ND
Seed	PL#S	3.89 ± 0.16 a
	PC#S	3.77 ± 0.12 a
Peel, mesocarp	PL#P	ND
	PC#P	ND

In each column, different letters mean statistically significant differences (*p* < 0.05) compared by the Tukey HSD test. ND: non-detected.

**Table 6:** Profile of phenolic compounds present in the analyzed pacay samples, and DPPH assay, determined by QUENCHER methodology (results expressed in dry weight, mean ± SD).

	Phenolic Compounds			Antioxidant Activity
Sample	Hydroxycinnamic Acids (mg FAE/100 g)	Flavonols (mg QE/100 g)	Total Phenolics (mg GAE/100 g)	DPPH (mg TE/g)
	Fruit edible fraction			
PL#F	43.55 ± 4.33 a	18.69 ± 1.69 a	332.55 ± 10.90 b	16.73 ± 0.29 b
PC#F	38.48 ± 3.76 a	21.50 ± 1.93 a	558.81 ± 22.10 c	13.60 ± 0.63 b
	Seed			
PL#S	371.48 ± 25.28 e	31.61 ± 2.95 b	204.31 ± 7.51 a	3.28 ± 0.25 a
PC#S	328.81 ± 26.20 d	47.54 ± 3.52 c	234.53 ± 6.91 a	4.72 ± 0.28 a
	Peel, mesocarp			
PL#P	125.86 ± 9.43 c	31.59 ± 2.96 b	1843.39 ± 82.59 e	27.58 ± 2.57 c
PC#P	101.97 ± 10.00 b	32.75 ± 3.11 b	1394.82 ± 111.65 d	41.58 ± 1.72 d

In each column, different letters mean statistically significant differences (*p* < 0.05) compared by the Tukey HSD test between varieties for each parameter and fruit fraction analyzed. FAE: ferulic acid equivalent; QE: quercetin equivalent; GAE: gallic acid equivalent; TE: Trolox equivalent.

## Data Availability

The original contributions presented in this study are included in the article. Further inquiries can be directed to the corresponding author.
